# Prenatal exposure to bisphenol A and phthalates and behavioral problems in children at preschool age: the Hokkaido Study on Environment and Children’s Health

**DOI:** 10.1186/s12199-018-0732-1

**Published:** 2018-09-07

**Authors:** Machiko Minatoya, Sachiko Itoh, Keiko Yamazaki, Atsuko Araki, Chihiro Miyashita, Naomi Tamura, Jun Yamamoto, Yu Onoda, Kazuki Ogasawara, Toru Matsumura, Reiko Kishi

**Affiliations:** 1 Center for Environmental and Health Sciences, Hokkaido University, Kita 12, Nishi 7, Kita-ku, Sapporo, 060-0812 Japan; 2 Institute of Environmental Ecology, IDEA Consultants, Inc., Shizuoka, Japan

**Keywords:** SDQ, Bisphenol A, Phthalates, Prenatal exposure, Birth cohort, Behavioral problems

## Abstract

**Background:**

Studies reported adverse behavioral development including internalizing and externalizing problems in association with prenatal exposure to bisphenol A (BPA) and phthalates; however, findings were not sufficient due to using different assessment tools and child ages among studies. This study aimed to examine associations between maternal serum levels of BPA and phthalate metabolites and behavioral problems at preschool age.

**Methods:**

The Strengths and Difficulties Questionnaire (SDQ) was used to assess behavioral problems at 5 years of age. BPA and phthalate metabolite levels in the first trimester maternal serum was determined by LC-MS/MS for 458 children. Variables used for adjustment were parental ages, maternal cotinine levels, family income during pregnancy, child sex, birth order, and age at SDQ completed.

**Results:**

The median concentrations of BPA, MnBP, MiBP, MEHP, and MECPP, primary and secondary metabolites of phthalates, were 0.062, 26.0, 7.0, 1.40, and 0.20 ng/ml, respectively. MECPP level was associated with increase conduct problem risk (OR = 2.78, 95% CI 1.36–5.68) overall and the association remained after child sex stratification, and odds ratios were increased with wider confidence interval (OR = 2.85, 95% CI 1.07–7.57 for boys, OR = 4.04, 95% CI 1.31–12.5 for girls, respectively). BPA, ∑DBP_m_ (MnBP + MiBP), and ∑DEHP_m_ (MEHP+MECPP) levels were not associated with any of the child behavioral problems.

**Conclusions:**

Our analyses found no significant association between BPA or summation of phthalate metabolite levels and any of the behavioral problems at 5 years of age but suggested possible association between MECPP levels and increased risk of conduct problems.

**Electronic supplementary material:**

The online version of this article (10.1186/s12199-018-0732-1) contains supplementary material, which is available to authorized users.

## Background

It has been reported that developmental disabilities have increased in recent decades [1, 2]. Childhood behavioral problems have influence on individual development, school performance and quality of life. Bisphenol A (BPA) and phthalates are ubiquitous environmental chemicals that were detected from various specimen including urine, blood, breast milk, and anomic fluid [3, 4]. BPA is widely used in polycarbonate products and epoxy resins as coatings on the inside of many food and beverage cans [5]. There are variety of phthalates used in consumer products such as food packages, polyvinyl chloride floor materials, lotion, and fragrances. Humans are exposed to phthalates by multiple routes. Exposures can be oral or dermal or can also be via inhalation [6]. Since BPA and phthalates can cross the placenta [7, 8], exposure during critical period in fetal development is a concern [6, 7].

Exposure to environmental chemicals such as bisphenol A (BPA) and phthalates may play roles in the development of child behavioral problems [9, 10]. BPA and phthalates are both known as endocrine disruptors and there is a growing concern of exposure to these chemicals and adverse health outcomes on human. From laboratory studies, BPA has been shown to disrupt brain function and structure [11–13].

Previously, several birth cohort studies have investigated associations between BPA and phthalate exposures and child behavioral problems. For example, maternal levels of BPA have been associated with various child behavioral outcomes including behavioral problems, internalizing and externalizing problems, cognitive development, anxiety, and so on in early childhood [14–20]. Maternal levels of phthalate including di-2-ethylhexyl phthalate (DEHP), butylbenzyl phthalate (BBzP), and dibutyl phthalates (DBP) were associated with adverse child neurodevelopmental outcomes including internalizing and externalizing problems; however, findings from these studies were inconsistent as the age of children at testing, testing tools, and outcomes varied from study to study [20–24]. Additionally, some of these studies found association only in specific child sex.

The present study examined the association of maternal levels of BPA and phthalates with child behavioral problems at preschool age using Strength and Difficulty Questionnaire (SDQ), a widely used assessment tool of child behavioral problems [25].

## Methods

### Study design and selection of study population

This study formed as a follow-up study of a prospective birth cohort study, the Hokkaido Study on Environment and Children’s Health. The details of cohort profile can be found elsewhere [26, 27]. In the Hokkaido Study, we established sub-cohort population and how we set it up was described as follows. The sub-cohort population consisted of 500 participants randomly selected from each enrollment year between 2003 and 2011, and all 369 participants from the enrollment year 2012. The total number of participants in sub-cohort population was 4869, which corresponded to 23.3% of all participants in the Hokkaido Study (*n* = 20,926). Establishing sub-cohort population is an effective way for avoiding the costs of collecting and processing covariate information and exposure assessment when a cohort is followed for various health outcomes.

**Fig. 1:**
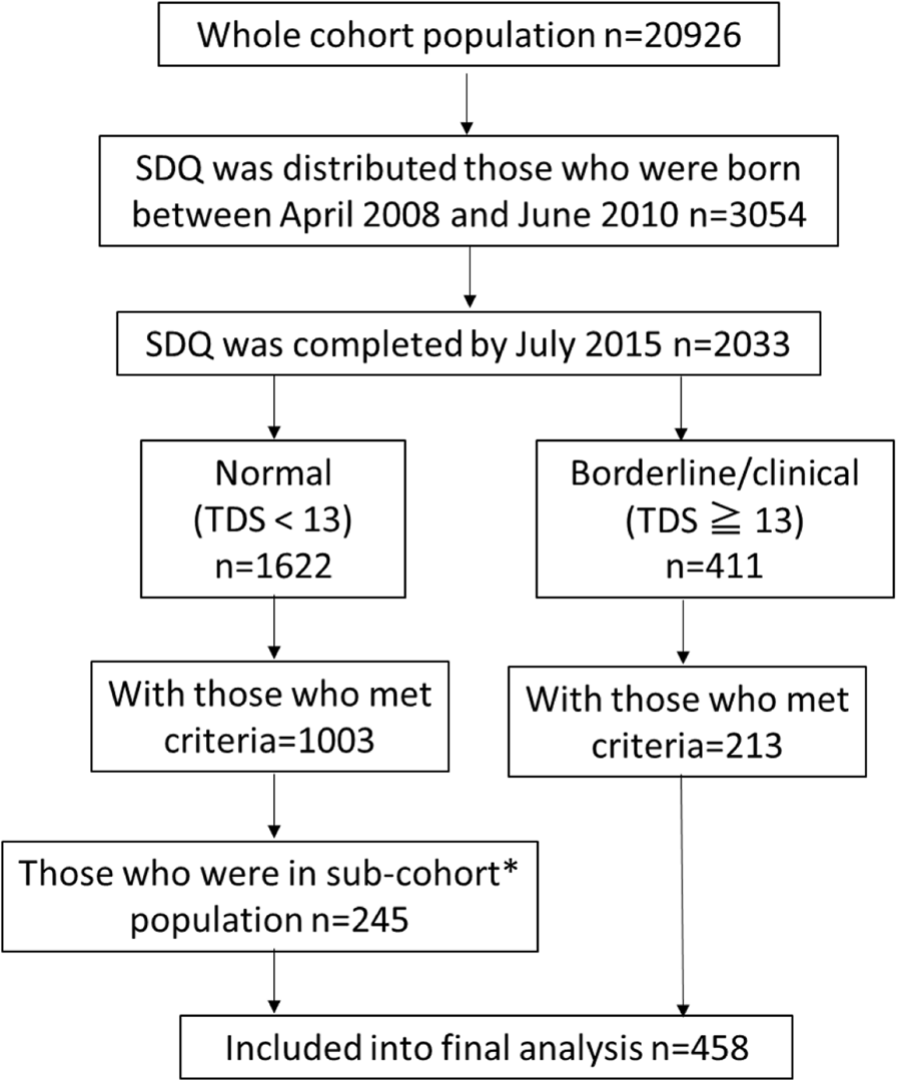
Selection of study population. *The sub-cohort of 4869 participants, which corresponded to 23.3% of all participants (*n* = 20,926) in the Hokkaido study were established. In this sub-cohort, 500 participants who were randomly selected from each enrollment year between 2003 and 2011, and all 369 participants from the enrollment year 2012 were included. The sub-cohort population was supposed to be representing original cohort population. The aim of establishing the sub-cohort population was for effective exposure assessments

In this particular follow-up study, the subpopulation consisted of all the cohort study participants who were born between April 2008 and June 2010 (*n* = 3054) out of all the participants (enrolled from 2003 to 2012, *n* = 90,926). Total 3054 SDQ were distributed via mail between October 2014 and June 2015 to the subpopulation. Two thousand thirty-three SDQ were successfully filled and returned by the end of July 2015 (response rate = 66.6%). Among 2033 children with valid completed SDQ, 1622 were classified into normal group and 411 were classified into borderline/clinical group based on total difficulties score of SDQ. Then we applied criteria for selecting participants to conduct exposure assessment. The criteria were follows: those who had maternal first trimester baseline questionnaire data, first and third trimester maternal blood samples, maternal and cord blood samples at delivery, birth record, follow-up questionnaires data at ages 1, 2, and 4 years of age to use as covariates. We decided to include all the children in borderline/clinical group (*n* = 213) as suspected cases and children those who met abovementioned criteria and those who were sub-cohort population (*n* = 245) as controls (Fig. 1).

### Assessment of child behavior

Japanese parent-report version of SDQ [28] were distributed via mail to the participants. Parents were asked to fill SDQ, which included 25 items on specific strengths and difficulties with an overall rating of whether their child had behavioral problems. SDQ was designed for a broad range of children, age 3 to 16 years and well validated tool of childhood mental health [25, 29]. Each item has three response categories: (0) not true, (1) somewhat true, and (3) certainly true. It includes five subscales (conduct problems, hyperactive/inattention, emotional problems, peer problems, and prosocial behavior). All subscale scores excluding prosocial behavior were summed as total difficulties score (ranged from 0 to 40 [29]) to assess the behavioral problems. Higher scores denote greater problems. We applied score bandings of the Japanese version of SDQ; children total difficulties with 0–12 were defined as normal, 13–15 were as borderline, and 16–40 were as clinical [28]. For the subscales, the following cut-offs were applied: *conduct problems*: 0–3 = normal, 4 = borderline, 5–10 = clinical; *hyperactivity/inattention*: 0–5 = normal, 6 = borderline, 7–10 = clinical; *emotional problems*: 0–3 = normal, 4 = borderline, 5–10 = clinical; *peer problems*: 0–3 = normal, 4 = borderline, 5–10 = clinical; *prosocial behavior*: 6–10 = normal, 5 = borderline, 0–4 = clinical [28]. SDQ total and subscale scores were dichotomized comparing the children with borderline and clinical scores with normal children.

### Exposure assessment

Concentrations of BPA and phthalate metabolites in the first trimester maternal serum samples of 213 cases and 245 controls were measured. Maternal serum of the first trimester was collected and stored at − 80 °C till analyses. Blood samples were analyzed for BPA and seven kinds of phthalate metabolites; mono-n-butyl phthalate (MnBP), mono-isobutyl phthalate (MiBP), mono-2-ethylhexyl phthalate (MEHP), mono-benzyl phthalate (MBzP), mono-2-ethyl-5-hydroxyhexyl phthalate (MEHHP), mono-2-ethyl-5-carboxypentyl phthalate (MECPP), and mono (4-methyl-7-carboxyheptyl) phthalate (cx-MiNP) by isotope-diluted liquid chromatography-tandem mass spectrometry (LC-MS/MS) for BPA analysis and ultra-performance LC-MS/MS for phthalate metabolites analysis. The method detection limits (MDLs) of BPA, MnBP, MiBP, MBzP, MEHP, MEHHP, MECPP, and cx-MiNP were 0.011, 0.57, 0.44, 0.19, 0.31, 0.23, 0.11, and 0.12 ng/ml, respectively. All the analyses were conducted at IDEA Consultants Inc. (Shizuoka, Japan). The detailed sample preparation for BPA analysis can be found from our previous report [30, 31]. Briefly to each serum sample, BPA-d_16_ β-glucuronidase spiking solution was added and shaken then β-glucuronidase and 0.2 M acetate buffer solution (pH 5.0) were added. Samples were held in an incubator at 37 °C for 1.5 h followed by solid phase extraction. The detailed phthalate metabolite analyses are described in our previous article [31]. For quality control, each batch of samples included five procedural blanks, two control samples, and repeated injections of an internal standard blank. The overall native BPA/BPA-d_16_ relative response factor (RRF) was − 10 to + 8.5%. The discrepancy in levels of BPA in duplicate measurements varied 0–23%, which were within the described standard range in the guidelines establishing test procedures for the analysis of pollutants by the Ministry of Environment of Japan [32]. The MDL of BPA was calculated as follows according to the procedure of the manual of Analyses of Chemicals by the Ministry of Environment of Japan [32] and was 0.011 ng/ml.

Briefly, serum samples for phthalate metabolite analyses were prepared as follows. MnBP-d_4_, MiBP-d_4_, MBzP-d_4_, MEHP-d_4_, MEHHP-^13^C_4_, MECPP-^13^C_4_, and cx-MiNP-d_4_ were added as surrogate and then 90 μL of 1 M phosphoric acid was added to the serum sample (0.5 mL). After mixing by vortex and ultrasonic irradiated for 10 min and consequently, 940 μL of acetonitrile was added and centrifuged with 3500 rpm for 5 min. Supernatants were transferred into new tubes and added 1000 μL of ammonium acetate buffer solution (100 mM, pH 9.1) and 3000 μL of ammonium acetate buffer solution (100 mM, pH 6.5), 10 μL of β-glucuronidase was added to each sample for the enzymatic hydrolysis of the phthalate metabolites conjugates, and 100 mM ammonium acetate solution was added. Samples were held in an incubator at 37 °C for 1.5 h followed by solid phase extraction by Oasis MAX 96 well plate (30 mg, 30 μm, Waters, Milford, MA, USA). After solid phase extraction, a 500 μL of elution was transferred into sample vials and added 500 μL of ultra-pure water and analyzed by UPLC (ACQUITY UPLC H-Class, Milford, MA, USA) coupled to triple quadrupole tandem MS (QTRAP 6500, AB SCIEX, Framingham, MA). The insoluble particulates were filtered by in-line filters (2.1 × 5 mm, 1.7 μm, Vanguard Phenyl column, Waters, Tokyo, Japan) preceding the BEH Phenyl column (2.1 × 50 mm, 1.7 μm, Waters, Tokyo, Japan). The retention gap technique was used by installing retention gap columns Atlantis T3 (2.1 × 50 mm, 3 μm, Waters, Tokyo, Japan), which improved phthalate metabolite sensitivity by trapping mobile-phase phthalate metabolites (contaminants) in the retention gap column. The column temperature was 40 °C. The total UPLC cycle time was 20 min including column re-equilibration. The calibration curve was linear over a concentration ranging from 0.02 to 20 ng/ml with a coefficient of correlation (*r*^2^) greater than 0.999. The procedural blank levels were determined using 0.5 mL of ultrapure water. The MDLs of BPA and phthalate metabolites were calculated as follows according to the procedure of the manual of Analyses of Chemicals by the Ministry of Environment of Japan [32].

### Covariates

Parental factors including ages, educational levels, maternal pre-pregnancy body mass index (BMI), parity, and family income were obtained from a baseline questionnaire which was filled by participants during their pregnancy. Additionally, maternal smoking status was examined from cotinine levels of third trimester maternal blood measured by using high-sensitive enzyme-linked immunosorbent assay (ELISA). The limit of detection (LOD) was 0.12 ng/ml. According to previous finding [33], we defined cotinine levels ≦ 0.21 ng/ml as non-smokers, 0.22–11.47 ng/ml as passive smokers, and ≧ 11.48 ng/ml as active smokers. Gestational age, birth weight, and gender of children were obtained from the birth record.

### Data analysis

Statistical analyses were performed using SPSS 22.0J (IBM Japan, Tokyo, Japan). Logistic regression models were used to calculate odds ratios (ORs) for having borderline/clinical scores (cases) in relation to maternal BPA and phthalate levels. The main analysis was case control study based on total difficulties scores. Then, subscales conduct problems, hyperactivity/inattention, emotional symptoms, peer problems, and prosocial behavior were investigated as sub-analyses. Maternal BPA and phthalate levels were log_10_ transformed and treated as continuous variables. The BPA and phthalate levels below MDL were replaced half the values of MDLs for statistical analyses. MEHP and MECPP were combined and expressed as the summation of DEHP metabolites (∑DEHP_m_). MEHHP was also a DEHP metabolite; however, in this study population, the detection rate was low, and thus, it was not included in the summation of DEHP metabolites. Similarly, MnBP and MiBP were combined and expressed as the summation of DBP metabolites (∑DBP_m_). To combine the metabolites, the summation of each metabolite expressed in molar concentration was multiplied with their respective parent molecular weight (MW) as follows:

$$ \sum {\mathrm{DEHP}}_{\mathrm{m}}=\left(\left({C}_{\mathrm{MEHP}}/{\mathrm{MW}}_{\mathrm{MEHP}}\right)+\left({C}_{\mathrm{MECPP}}/{\mathrm{MW}}_{\mathrm{MECPP}}\right)\right)\ast {\mathrm{MW}}_{\mathrm{DEHP}} $$

$$ \sum {\mathrm{DBP}}_{\mathrm{m}}=\left(\left({C}_{\mathrm{MnBP}}/{\mathrm{MW}}_{\mathrm{MnBP}}\right)+\left({C}_{\mathrm{MiBP}}/{\mathrm{MW}}_{\mathrm{MiBP}}\right)\right)\ast {\mathrm{MW}}_{\mathrm{DBP}} $$

where *C* is the measured concentration (ng/ml) and MW is the molecular weight (ng/nmol).

The ORs were given for one-unit increase on log_10_ scale. Covariate included in the final models were identified a priori using directed acyclic graph: parental ages (continuous), maternal cotinine levels (≦ 0.21 ng/ml vs. 0.22–11.47 ng/ml vs. ≧ 11.48 ng/ml), family income during pregnancy (< 5 M vs. ≧ 5 M) and birth order (first vs. not first). In addition to the abovementioned covariates, we included child sex and child age (months) at SDQ completed in the models based on previous literature. Further analysis was conducted for stratification of child sex. To control the familywise error rate caused by multiple comparisons in logistic regression analyses, Bonferroni collection was applied and *p* value of < 0.01 was considered statistically significant.

For the sensitive analysis, we used 80% cutoff scores of SDQ total and subscales. The results for the sensitive analysis was presented in Additional file 1: Table S4.

## Results

Table 1 shows the comparison of characteristics of participants in two groups (normal vs. borderline/clinical). Both maternal and paternal ages were younger in borderline/clinical group compared to normal group. Maternal pre-pregnancy body mass index (BMI) was higher in borderline/clinical group. Percentage of family income during pregnancy < 5 million Japanese Yen was higher in borderline/clinical group. Percentage of maternal cotinine level ≧ 11.48 ng/ml (active smokers) was higher in borderline/clinical group. Child characteristics including gestational age, birth weight, and age at SDQ completed were not different between two groups. The percentages of being first child and boy gender were higher in borderline/clinical group.

**Table 1:** Basic characteristics of parents and their children

Characteristics		Normal (*n* = 245)	Borderline/clinical (*n* = 213)	*p* value
Maternal age (years)		31.5 ± 4.3	29.8 ± 4.8	< 0.001
Paternal age (years)		33.4 ± 5.5	31.3 ± 5.1	< 0.001
Maternal pre-pregnancy BMI (kg/m^2^)		20.7 ± 2.5	21.4 ± 3.3	0.020
0.02 maternal cotinine levels at third trimester (ng/ml)	≦ 0.21 (non-smoker)	151 (61.6)	97 (45.5)	< 0.001
	0.22–11.47 (passive smoker)	81 (33.1)	93 (43.7)	
	≧ 11.48 (active smoker)	13 (5.3)	23 (10.8)	
Maternal education (years)	≦ 12	88 (35.9)	92 (43.2)	0.107
	≧ 13	154 (62.8)	118 (55.4)	
	Missing	3 (1.2)	3 (1.4)	
Paternal education (years)	≦ 12	89 (36.3)	86 (40.4)	0.326
	≧ 13	154 (62.9)	123 (57.7)	
	Missing	2 (0.8)	4 (1.9)	
Family income during pregnancy (JPY)	< 5 M	125 (51.0)	133 (62.4)	0.001
	≧ 5 M	90 (36.7)	48 (22.5)	
	Missing	30 (12.2)	32 (15.0)	
Family income at SDQ completed (JPY)	< 5 M	111 (45.3)	111 (52.1)	0.008
	≧ 5 M	123 (50.2)	88 (41.3)	
	Missing	11 (4.5)	14 (6.6)	
Marital Status at SDQ completed	Married	236 (96.3)	198 (93.0)	0.116
Gestational age (days)		275.3 ± 8.2	275.4 ± 8.5	0.897
Birth weight (g)		3037 ± 339	3076 ± 383	0.240
Child sex	Boy	122 (49.8)	128 (60.1)	0.027
	Girl	123 (50.2)	85 (39.9)	
Birth order	First child	116 (47.3)	123 (57.7)	0.026
Age at SDQ completed (months)		67.3 ± 6.2	66.3 ± 6.3	0.099

Mean ± S.D. or *n* (%)

*JPY* Japanese Yen

Table 2 presents distribution of BPA and phthalate metabolite levels in maternal blood of all of three groups. The detection rates of BPA, MnBP, MiBP, MBzP, MEHP, MEHHP, MECPP, and cx-MiNP were 94.0%, 100.0%, 100.0%, 9.1%, 96.5%, 0.7%, 82.1%, and 0.4%, respectively. The detection rates of MBzP, MEHHP, and cx-MiNP were below 10%. Thus, these chemicals were excluded from the further analyses. The median concentration of BPA was significantly different among the three groups and borderline and clinical groups showed higher levels compared to the normal group (*p* = 0.049). All the other concentrations were not significantly different among the three groups.

**Table 2:** Comparison of the distribution of BPA and phthalate metabolite levels in maternal blood of the three groups

Exposure	MDL (ng/ml)	Detection rate (%)	Normal (*n* = 245)		Borderline (*n* = 125)		Clinical (*n* = 88)		*p* value*
			Median	IQR	Median	IQR	Median	IQR	
BPA	0.011	94.0	0.054	0.022, 0.207	0.074	0.027, 0.370	0.097	0.037, 0.320	0.049
MnBP	0.57	100.0	26.7	17.7, 37.6	25.1	16.8, 34.0	24.0	17.0, 35.0	0.221
MiBP	0.44	100.0	7.4	5.3, 9.9	6.6	4.7, 8..7	7.2	5.3, 9.1	0.166
MBzP	0.19	9.1	<MDL	<MDL, <MDL	<MDL	<MDL, <MDL	<MDL	<MDL, <MDL	N/A
MEHP	0.31	96.5	1.42	0.82, 9.07	1.25	0.78, 8.20	1.53	067, 10.00	0.644
MEHHP	0.23	0.7	<MDL	<MDL, <MDL	<MDL	<MDL, <MDL	<MDL	<MDL, <MDL	N/A
MECPP	0.11	82.1	0.20	0.11, 0.30	0.21	0.11, 0.32	0.21	0.13, 0.34	0.470
cx-MiNP	0.12	0.4	<MDL	<MDL, <MDL	<MDL	<MDL, <MDL	<MDL	<MDL, <MDL	N/A

*MDL* method detection limit, *IQR* interquartile range

*Kruskal-Wallis test

Table 3 presents adjusted odds ratios for a tenfold increase of maternal BPA and individual and summation of DBP and DEHP metabolite levels on having behavioral problems. MECPP level was significantly associated with an increased risk of conduct problems (OR = 2.78, 95% confidence interval (CI) 1.36–5.68). This association remained after child sex stratification, and odds ratios were increased with wider confidence interval (OR = 2.85, 95% CI 1.07–7.57 for boys, OR = 4.04, 95% CI 1.31–12.5 for girls, respectively). MECPP level was also associated with an increased risk of hyperactivity/inattention among girls with a wide CIs (OR = 5.71, 95% CI 1.41–23.1). There were no significant association between∑DBP_m_ and ∑DEHP_m_ levels and any of the behavioral problem risks. BPA level was associated with an increased risk of prosocial behavior (OR = 1.46, 95% CI 1.04–2.06) overall and the association was stronger among boys (OR = 1.68, 95% CI 106, 2.68) compared to girls (OR = 1.31, 95% CI 0.77, 2.24).

**Table 3:** Adjusted odds ratios for a tenfold increase of maternal BPA and phthalate metabolite levels on having behavioral problems

		Number of children in borderline/clinical	BPA	MnBP	MiBP	MEHP	MECPP	∑DBP_m_	∑DEHP_m_
			OR (95% CI)						
All	Total difficulties (≧ 13)	213	1.28 (0.94, 1.74)	0.51 (0.22, 1.18)	0.42 (0.17, 1.03)	0.93 (0.65, 1.33)	1.13 (0.58, 1.13)	0.48 (0.20, 1.13)	0.93 (0.63, 1.38)
	Conduct problems (≧ 4)	142	1.15 (0.84, 1.58)	1.33 (0.55, 3.20)	1.37 (0.53, 3.58)	0.81 (0.56, 1.18)	2.78 (1.36, 5.68)*	1.34 (0.54, 3.33)	0.82 (0.55, 1.24)
	Hyperactivity/inattention (≧ 6)	126	1.06 (0.75, 1.51)	1.10 (0.42, 2.84)	0.93 (0.33, 2.65)	1.22 (0.82, 1.84)	1.52 (0.71, 3.29)	1.04 (0.39, 2.80)	1.25 (0.81, 1.95)
	Emotional symptoms (≧ 4)	116	0.92 (0.66, 1.27)	0.83 (0.35, 1.98)	0.57 (0.22, 1.45)	0.86 (0.59, 1.27)	0.65 (0.33, 1.31)	0.77 (0.31, 1.88)	0.85 (0.56, 1.28)
	Peer problems (≧ 4)	64	0.99 (0.65, 1.52)	0.92 (0.30, 2.87)	0.45 (0.14, 1.49)	0.78 (0.47, 1.29)	0.90 (0.36, 2.25)	0.79 (0.25, 2.54)	0.76 (0.44, 1.44)
	Prosocial behavior (≦ 5)	112	1.46 (1.04, 2.06)^+^	0.97 (0.39, 2.40)	0.96 (0.36, 2.56)	0.89 (0.59, 1.33)	1.10 (0.53, 2.31)	0.95 (0.37, 2.42)	0.88 (0.56, 1.36)
Boy	Total difficulties (≧ 13)	128	1.26 (0.82, 1.95)	0.54 (0.18, 1.61)	0.43 (0.13, 1.46)	0.82 (0.49, 1.35)	0.62 (0.24, 1.60)	0.50 (0.16, 1.58)	0.79 (0.46, 1.37)
	Conduct problems (≧ 4)	83	1.32 (0.86, 2.03)	1.14 (0.36, 3.55)	0.95 (0.27, 3.30)	0.79 (0.48, 1.31)	2.85 (1.07, 7.57)^+^	1.09 (0.33, 3.56)	0.78 (0.45, 1.36)
	Hyperactivity/inattention (≧ 6)	85	0.80 (0.50, 1.28)	1.03 (0.32, 3.32)	0.87 (0.24, 3.14)	1.05 (0.63, 1.76)	0.92 (0.35, 2.44)	0.98 (0.29, 3.31)	1.06 (0.61, 1.85)
	Emotional symptoms (≧ 4)	77	0.89 (0.56, 1.42)	0.78 (0.24, 2.53)	0.52 (0.14, 1.86)	0.97 (0.57, 1.63)	0.65 (0.24, 1.75)	0.71 (0.21, 2.43)	0.95 (0.54, 1.68)
	Peer problems (≧ 4)	40	0.96 (0.54, 1.72)	0.74 (0.17, 3.32)	0.50 (0.10, 2.53)	0.67 (0.34, 1.31)	0.68 (0.20, 2.37)	0.67 (0.14, 3.18)	0.64 (0.31, 1.33)
	Prosocial behavior (≦ 5)	73	1.68 (1.06, 2.68)^+^	0.72 (0.24, 2.22)	0.65 (0.19, 2.25)	1.03 (0.62, 1.74)	1.19 (0.45, 3.11)	0.69 (0.21, 2.21)	1.04 (0.59, 1.83)
Girl	Total difficulties (≧ 13)	85	1.30 (0.83, 2.03)	0.26 (0.06, 1.06)	0.25 (0.06, 1.09)	1.10 (0.64, 1.88)	2.37 (0.87, 6.42)	0.24 (0.06, 1.03)	1.16 (0.65, 2.08)
	Conduct problems (≧ 4)	59	1.03 (0.63, 1.67)	0.90 (0.19, 4.16)	1.46 (0.29, 7.40)	0.91 (0.50, 1.63)	4.04 (1.31, 12.5)^+^	0.98 (0.20, 4.78)	0.96 (0.51, 1.82)
	Hyperactivity/inattention (≧ 6)	41	1.66 (0.95, 2.90)	1.05 (0.17, 6.38)	0.95 (0.14, 6.50)	1.68 (0.84, 3.37)	5.71 (1.41, 23.1)^+^	0.99 (0.15, 6.41)	1.79 (0.84, 3.81)
	Emotional symptoms (≧ 4)	39	0.93 (0.57, 1.51)	0.45 (0.10, 1.95)	0.34 (0.07, 1.64)	0.77 (0.43, 1.37)	0.84 (0.30, 2.33)	0.41 (0.09, 1.86)	0.76 (0.41, 1.41)
	Peer problems (≧ 4)	24	1.08 (0.56, 2.09)	0.64 (0.09, 4.66)	0.18 (0.02, 1.33)	1.06 (0.47, 2.39)	1.24 (0.30, 5.20)	0.47 (0.06, 3.54)	1.09 (0.46, 2.62)
	Prosocial behavior (≦ 5)	39	1.31 (0.77, 2.24)	1.60 (0.31, 8.29)	1.76 (0.32, 9.76)	0.66 (0.33, 1.31)	0.78 (0.23, 2.69)	1.62 (0.30, 8.75)	0.63 (0.30, 1.33)

Adjusted for parental ages, maternal cotinine levels, family income during pregnancy, child sex, birth order (first child or not), and child age at SDQ complete. * *p* < 0.01, +*p* < 0.05

## Discussion

Recent reviews have shown that environmental chemicals may play a role in the etiology of behavioral and developmental disorders [34, 35]. In our study, prenatal exposure to BPA and phthalates was measured in maternal blood of first trimester and child behavioral problems at 5 years of age were assessed using the SDQ. Our analyses found no significant association between summation of phthalate metabolite levels and an increased risk of any of the behavioral problems at 5 years of age but suggested possible association between BPA levels and increased risk of prosocial behavior and between MECPP levels and increased risk of conduct problems. The sensitive analysis using 80% cutoff scores of SDQ found basically no change in findings; however, the significance was slightly attenuated (Additional file 1: Table S4). Stratification by child sex analyses found that maternal MECPP level was associated with an increased risk of hyperactivity/inattention problems only in girls with a large confidence interval. This could be because the number of individuals was too small in some categories of the adjustment factors, since the crude model found no statistical significance (OR = 1.32, 95% CI 0.70–2.48). Thus, the interpretation of findings from the adjusted model should be carried out cautiously.

SDQ score of 2033 children in this study was 8.7 and was similar to the other previous studies in the UK (5–10 years old) and Japan (4–6 years old), which showed average scores of 8.3 and 8.6, respectively [28, 36]. The BPA level in this study was of a similar range to a previous report of Japanese pregnant women [30] and lower compared to that of pregnant women in other studies [37–39].

There have been several prospective cohort studies that investigated associations between prenatal exposure to BPA and child behavioral problems [14, 15, 17–20, 40–43]. Our group assessed child behavioral problems at 3.5 years of age using Child Behavior Checklist (CBCL) and found that cord blood BPA level was positively associated with internalizing problem and development problem scores [43]. Braun et al. assessed child behavior at different ages using the prospective birth cohort in the US (HOME Study) [17, 18, 40]. In their study, among girls, higher maternal urinary BPA was associated with increased aggression and hyperactivity at age 2 [17]. The follow-up of the same cohort at 3 years of age found that higher maternal urinary BPA was associated with more anxiety and depression of Behavioral Assessment System for Children-Second Edition (BASC-2) and poorer emotional control of Behavior Rating Inventory of Executive Function-Preschool (BREIF-P) only among girls [18]. In our study, we did not find the statistical significance; however, increased odds of hyperactivity/inattention among girls in association with increased BPA level were consistent with the findings from Braun et al. [17, 18]. Another birth cohort study in the US (CCCEH) also investigated association between maternal urinary BPA and child behavior [14, 42]. The results of their study showed that higher levels of maternal BPA were associated with higher scores on emotionally reactive and aggressive behavior subscales of CBCL among boys at 5 years of age [14]. A follow-up of the same cohort at 7–9 years of age found that higher maternal BPA levels were associated with more anxiety and depression in boys [42]. Harley et al. investigated association between maternal urinary BPA and school-aged child behavior in the birth cohort study (CHAMACOS) [15]. They found that higher maternal BPA was associated with higher depression and anxiety in boys. Evans et al. reported that higher maternal BPA was associated with higher level of aggression, anxiety, oppositional/defiant problems, and conduct problems in boys using CBCL at ages 6–10 years in a birth cohort study (SFF II) [44]. Most of the previous studies found sex-specific effects of BPA exposure on child behavioral development and problems, and we also found stronger association on prosocial behavior in boys compared to girls. Inconsistent findings from the previous studies could be due to different exposure assessment timings among studies. The critical period of exposure to BPA during pregnancy on child neurobehavioral development is still not evident, thus using maternal blood samples of the first trimester may not well evaluate associations between prenatal exposures and outcomes. Braun et al. reported a relationship between maternal urinary BPA and child behavior, and the relationship was stronger with urine samples of ≦ 16 weeks of gestation compared to that of 26 weeks of gestation, which suggested a possible critical period for BPA exposure on neurobehavior development [17]. Our result indicated that the first trimester BPA level was associated with increased risk of hyperactivity/inattention among girls without significance, which is in line with the previous findings [17]. Further investigation is required to elucidate critical exposure period of BPA exposure and its influence on child behavioral development.

Various study population background may also be a reason for inconsistent findings. For example, maternal education levels > high school in this study was 62.8%, whereas it varied from low to high (21.6% [15] to 85% [44]) in the previous studies that found association between BPA exposure and child behavioral problems. It has been reported that lower maternal education can be a predictor of higher BPA levels [18, 45]. Thus, it may have contributed to inconsistent findings. Similarly, income is inversely associated with BPA levels according to NHANES data [46], and thus, different cultural backgrounds such as poverty rate and ethnicity could be a reason for inconstancy. Overall, the study population in this study was relatively well educated and tended to be less poverty, which may contribute to a different finding from previous studies.

There have been several reports from birth cohort studies regarding child behavioral development in association with prenatal phthalate exposure. Results from birth cohort studies have suggested that low molecular weight (LMW) phthalate such as DBP and DEP exposures might increase behavioral problems [20, 21, 41, 47]. Whyatt et al. assessed child behavioral problems using CBCL at 3 years old in association with maternal urine phthalate levels [21]. In their study, MnBP, MiBP, and MBzP were found to be associated with increased behavioral problems. However, no association was found between maternal urinary DEHP metabolites and child behavioral problems. Engel et al. investigated associations between maternal phthalate metabolites and child behavior at 4–9 years old using Behavior Assessment System for Children-Parent Rating Scale (BASC-PRS) [47]. Increased levels of LMW phthalate metabolites were associated with various behavioral problems including aggression, conduct problems, attention problems, and depression. The same group also used Social Responsiveness Scale (SRS) to assess child behavior at ages 7–9 years of age [41]. It was found that LMW phthalates were also associated with poorer social cognition, social communication, and social awareness. In our study, we did not find any association between LMW phthalates and child behavioral problems. Kobrosly et al. examined child neurobehavior using CBCL among children at 6–10 years of age [22]. They found increased third trimester maternal urine MiBP was associated with attention problems and aggressive behavior and the association was mostly observed among boys. Lien et al. assessed child behavior at 8–9 years of age using CBCL [23]. In their study, third trimester maternal MBP and MEOHP were associated with delinquent behavior and aggressive behavior scores at 8 years old. Recently, Gascon et al. assessed child behavioral problems using CBCL at 4 and SDQ at 7 years in the INMA-Sabadell birth cohort study [48]. They found that the average concentrations of the sum of four kinds of DEHP metabolites (MEHHP, MEHP, MEOHP, and MECPP) in maternal urine of first and third trimesters were associated with increased social competence scores at 4 years. Contrarily, they found that MEP concentrations were associated with a reduced risk of inattention symptoms at 4 years. One previous study reported that maternal MECPP level was inversely associated with child motor development at age 24–36 months only in girls [49]. In their study, not only MECPP but also the sum of DEHP metabolites and other DEHP metabolites (MEHHP, MEHP, MEOHP) were negatively associated with child motor development, which was inconsistent with our results. Overall, our findings from this study were not in line with these previous studies, as most of the studies reported effects of LMW phthalate exposures.

It should be mentioned that there were some protective effects of prenatal exposure to phthalates on child behavioral problems even though it did not reach statistical significance. This might be explained by the possibility that other unmeasured factors such as nutrition and living environment that were strongly correlated with maternal phthalate levels were actually associated with child behavioral problems.

A number of factors including assessment tools for outcome measurements and age at assessment, timing of exposure assessment, and genetic and demographic variety of study populations, as well as other unknown factors could explain the inconstancies among studies. Different levels of exposure among studies could also explain the different findings. Most of the previous studies used maternal urine samples during pregnancy for exposure assessment, whereas we used maternal serum. Even though a study reported correlation between serum and urine MECPP levels [50], direct comparison of exposure levels with other studies were not possible. It also should be noted that measurable levels are much higher in urine compared to blood samples for BPA and phthalate metabolites and that single time point serum samples are not considered as reliable as 24-h collected urine samples for assessment of internal exposure to BPA. Regarding BPA measurement using blood samples, it can possibly be overestimated due to external contamination. In this study, we used glass cartridge to reduce background levels and no free BPA was detected [30], which was an indication of null possible external contamination. Additionally, background level was measured and confirmed that the influence of external contamination was null. Hydrolytic enzymes are present in blood samples and may be responsible for diester to monoester conversion after the blood sample is drawn [51]. Analysis of monoester may yield higher levels because of monoester conversion of ex vivo contamination during the sampling, storage, and handling process. To minimize the influence of enzyme activity, the blood samples were immediately stored at − 80 °C and acid was added immediately after thawing. We still cannot rule out possible external contamination during the process of sample drawing, storage, and measurement. Using secondary metabolites of phthalates was recommended. In this study, we found behavioral problems in association with MECPP, which is a secondary metabolite of DEHP.

Limitations of this study should also be discussed. First, our exposure assessment was based on the single measurement which could not represent exposure of entire pregnancy period due to short half-lives of BPA and phthalates. Thus, the critical period of exposure might not be well captured in this study. Other limitation was that we had no information on factors that might have influence on the outcomes such as family psychopathology and exposure to psychosocial environmental stressors. There might be unmeasured or uncontrolled factors which possibly related to maternal BPA and phthalate exposure levels as well. This study used parent-reported SDQ scores, which may not be sufficient because the answers may have been hindered by a mood or mental condition of a parent. Further study using SDQ reported from other than parents should be considered. Sample size can be another limitation of this study especially in sex-specific analyses. Some of the subscales of SDQ showed a small number of children in borderline/clinical group (Additional file 1: Table S3). A wide range of 95% CIs observed in sex-stratification analyses indicated that the sample size was too small. It also should be noted that there might be a chance that associations may possibly be identified due to the number of chemicals tested even though we adjusted threshold for multiple comparison.

It should be noted that we did not measure postnatal exposures in this study. Some of the cross-sectional and birth cohort studies reported associations between postnatal exposure to BPA or phthalate exposures and child neurobehavioral development [52–57]. However, two of the prospective studies revealed that only gestational but not childhood BPA was associated with child behavior [14, 18]. Thus, we considered effects of prenatal exposure were more influential on child behavioral development.

The characteristics of participants in this study (*n* = 458), those who completed SDQ (*n* = 2033) and whole cohort population (*n* = 18,935), were compared in Additional file 1: Table S1. Parental education levels were relatively higher, and the percentage of maternal smoking was relatively lower in those who completed SDQ compared to the whole cohort population; this may indicate that parents with higher education levels are more concerned of their child’s behavioral development and can be a selection bias. Population in this study (*n* = 458) and those who completed SDQ (*n* = 2033) did not show substantial differences in their characteristics such as maternal pre-pregnancy BMI, parental ages, parental education, and family income. The percentage of non-smokers based on maternal cotinine levels was slightly higher in those included in this study; however, the percentages of active smokers were similar between the two groups. Finally, characteristics of participants in this study (*n* = 245) and those who completed (*n* = 1622) in the normal category were compared (Additional file 1: Table S2). Similarly, characteristics of the two groups in borderline/clinical category were compared (Additional file 1: Table S2). In both groups, participants in this study and those who completed SDQ showed similar distribution in characteristics. Thus, they are considered to be null or a small selection bias existed between those included in this study and not included.

## Conclusions

We found no significant association between BPA or summation of phthalate metabolite levels and any of the behavioral problems of children at preschool age but suggest possible association between MECPP levels and increased risk of conduct problems.

## Additional file

Additional file 1: **Table S1.** Characteristics of participants in this study (*n* = 458), those who completed SDQ (*n* = 2033) and whole cohort population (*n* = 18,931, live-birth only). **Table S2.** Comparison of characteristics of participants in this study and who completed SDQ stratified by SDQ total difficulties categories. **Table S3.** SDQ score distribution stratified by child sex. **Table S4.** Adjusted odds ratios for a tenfold increase of maternal BPA and phthalate metabolite levels on having behavioral problems (total difficulties) among 3 groups (normal, borderline, and clinical). **Table S5.** Adjusted odds ratios for a tenfold increase of maternal BPA and phthalate metabolite levels on having behavioral problems using 80% cutoff scores. (DOCX 29 kb)

## Abbreviations

BASC-2: Behavioral Assessment System for Children-Second Edition
BBzP: Butylbenzyl phthalate
BMI: Body mass index
BPA: Bisphenol A
BREIF-P: Behavior Rating Inventory of Executive Function-Preschool
CBCL: Child Behavior Checklist
CI: Confidence interval
cx-MiNP: Mono (4-methyl-7-carboxyheptyl) phthalate
DBP: Dibutyl phthalates
DEHP: Di-2-ethylhexyl phthalate
ELISA: Enzyme-linked immunosorbent assay
LC-MS/MS: Isotope-diluted liquid chromatography-tandem mass spectrometry
LOD: Limit of detection
MBzP: Mono-benzyl phthalate
MDLs: Method detection limits
MECPP: Mono-2-ethyl-5-carboxypentyl phthalate
MEHHP: Mono-2-ethyl-5-hydroxyhexyl phthalate
MEHP: Mono-2-ethylhexyl phthalate
MiBP: Mono-isobutyl phthalate
MnBP: Mono-n-butyl phthalate
MW: Molecular weight
ORs: Odds ratios
RRF: Relative response factor
SDQ: Strengths and Difficulties Questionnaire
SRS: Social Responsiveness Scale
