# Supplementary material for: Prenatal exposure to bisphenol A and phthalates and behavioral problems in children at preschool age: the Hokkaido Study on Environment and Children’s Health
Source: Environ Health Prev Med. 2018 Sep 7;23:43. doi: 10.1186/s12199-018-0732-1 (PMC6129008; doi:10.1186/s12199-018-0732-1)
Supplement: Supplementary file 1 — Table S1. Characteristics of participants in this study (n = 458), those who completed SDQ (n = 2033) and whole cohort population (n = 18,931, live-birth only). Table S2. Comparison of characteristics of participants in this study and who completed SDQ stratified by SDQ total difficulties categories. Table S3. SDQ score distribution stratified by child sex. Table S4. Adjusted odds ratios for a tenfold increase of maternal BPA and phthalate metabolite levels on having behavioral problems (total difficulties) among 3 groups (normal, borderline, and clinical). Table S5. Adjusted odds ratios for a tenfold increase of maternal BPA and phthalate metabolite levels on having behavioral problems using 80% cutoff scores. (DOCX 29 kb) [file 12199_2018_732_MOESM1_ESM.docx]

Table S1 Characteristics of participants in this study (n=458), those who completed SDQ (n=2033) and whole cohort population (n=18935, live-birth only).

| **Characteristics** | | **n （%）or mean ± S.D.** | | |
| --- | --- | --- | --- | --- |
|  |  | This study (n=458) | Who completed SDQ (n=2033) | Whole cohort population (n=18935) |
| **Maternal age（years）** |  | 30.7 ± 4.6 | 31.4 ± 4.6 | 30.4 ± 4.8 |
| **Maternal education** | **Middle school** | 13 (2.8) | 71 (3.5) | 979 (5.2) |
|  | **High School** | 167 (36.5) | 733 (36.1) | 7862 (41.5) |
|  | **Community college, vocational school** | 203 (44.4) | 876 (43.1) | 7281 (38.5) |
|  | **College or above** | 69 (15.1) | 304 (15.0) | 1958 (10.3) |
| **Maternal BMI（kg/m^2^）** |  | 21.0 ± 3.0 | 21.1 ± 3.3 | 21.2 ± 3.4 |
| **Parity** | **≧ 1** | 222 (48.6) | 1067 (52.8) | 9831 (51.9) |
| **Maternal cotinine levels (ng/ml)** | **≦ 0.21 (non-smokers)** | 248 (54.3) | 933 (45.9) | 6253 (33.0) |
|  | **0.22-11.48 (passive smokers)** | 174 (38.1) | 646 (31.8) | 7122 (37.6) |
|  | **> 11.48 (active smokers)** | 36 (7.9) | 162 (8.0) | 2372 (12.5) |
| **Family income**  **(million yen/year)** | **< 3** | 86 (18.8) | 354 (17.4) | 3547 (18.7) |
|  | **3-5** | 172 (37.6) | 756 (37.2) | 6906 (36.5) |
|  | **5-8** | 103 (22.5) | 516 (25.4) | 3918 (20.7) |
|  | **≧ 8** | 35 (7.7) | 143 (7.0) | 1154 (6.1) |
| **Paternal age（years）** |  | 32.5 ± 5.5 | 33.0 ± 5.5 | 32.2 ± 5.7 |
| **Paternal education** | **Middle school** | 16 (3.5) | 97 (4.8) | 1394 (7.4) |
|  | **High School** | 166 (36.3) | 759 (37.3) | 7477 (39.5) |
|  | **Community college, vocational school** | 135 (29.5) | 487 (24.0) | 4179 (22.1) |
|  | **College or above** | 142 (31.1) | 630 (31.0) | 4769 (25.1) |
| **Infant sex** | **Boy** | 250 (54.6) | 1007 (49.6) | 9552 (50.5) |
| **Gestational age (days)** |  | 275 ± 8 | 274 ± 11 | 274 ± 11 |
| **Birth weight　（g）** |  | 3055 ± 361 | 3005 ± 419 | 3019 ± 432 |

Table S2 Comparison of characteristics of participants in this study and who completed SDQ stratified by SDQ total difficulties categories.

| **Characteristics** | | **n （%）or mean ± S.D.** | | | |
| --- | --- | --- | --- | --- | --- |
|  | | **Normal** | | **Borderline/clinical** | |
|  |  | **this study (n=245)** | **who completed SDQ (n=1622)** | **this study (n=213)** | **who completed SDQ (n=411)** |
| **Maternal age（years）** |  | 31.5 ± 4.3 | 31.6 ± 4.5 | 29.8 ± 4.8 | 30.6 ± 4.9 |
| **Maternal education** | **≦ 12** | 88 (35.9) | 604 (37.2) | 92 (43.2) | 200 (48.7) |
|  | **≧ 13** | 154 (62.8) | 979 (60.4) | 118 (55.4) | 201 (48.9) |
| **Maternal BMI（kg/m^2^）** |  | 21.0 ± 3.0 | 21.0 ± 3.2 | 21.2 ± 3.4 | 21.6 ± 3.7 |
| **Parity** | **≧ 1** | 132 (53.9) | 877 (54.1) | 90 (42.3) | 190 (46.2) |
| **Maternal cotinine levels (ng/ml)** | **≦ 0.21 (non-smokers)** | 151 (61.6) | 776 (47.8) | 97 (45.5) | 157 (38.2) |
|  | **0.22-11.48 (passive smokers)** | 81 (33.1) | 510 (31.4) | 93 (43.7) | 136 (33.1) |
|  | **> 11.48 (active smokers)** | 13 (5.3) | 119 (7.3) | 23 (10.8) | 43 (10.5) |
| **Family income**  **(million yen/year)** | **< 5M** | 125 (51.0) | 857 (52.9) | 133 (62.4) | 253 (61.6) |
|  | **≧ 5M** | 90 (36.7) | 558 (34.4) | 48 (22.5) | 101 (24.6) |
| **Paternal age（years）** |  | 32.5 ± 5.5 | 33.3 ± 5.4 | 32.2 ± 5.7 | 32.0 ± 5.5 |
| **Paternal education** | **≦ 12** | 89 (36.3) | 670 (41.3) | 86 (40.4) | 186 (45.3) |
|  | **≧ 13** | 154 (62.9) | 907 (55.9) | 123 (57.7) | 20 (51.1) |
| **Infant sex** | **Boy** | 122 (49.8) | 758 (46.7) | 128 (60.1) | 248 (60.3) |
| **Gestational age (days)** |  | 275 ± 8 | 274 ± 11 | 275 ± 9 | 273 ± 13 |
| **Birth weight　（g）** |  | 3037 ± 339 | 3006 ± 409 | 3076 ± 383 | 3002 ± 429 |

Table S3 SDQ score distribution stratified by child sex.

|  | Boys (n=250) | | Girls (n=208) | |
| --- | --- | --- | --- | --- |
|  | Normal | Borderline/clinical | Normal | Borderline/clinical |
| **Total difficulties** | 122 (48.8) | 128 (51.2) | 123 (59.1) | 85 (40.9) |
| **Conduct problems** | 167 (66.8) | 83 (33.2) | 149 (71.6) | 59 (28.4) |
| **Hyperactivity/inattention** | 165 (66.0) | 85 (34.0) | 167 (80.3) | 41 (19.7) |
| **Emotional symptoms** | 173 (69.2) | 77 (30.8) | 169 (81.3) | 39 (18.7) |
| **Peer problems** | 210 (84.0) | 40 (16.0) | 184 (88.5) | 24 (11.5) |
| **Prosocial behavior** | 177 (70.8) | 73 (29.2) | 169 (81.2) | 39 (18.8) |

n (%).

Table S4 Adjusted odds ratios for ten folds increase of maternal BPA and phthalate metabolite levels on having behavioral problems (total difficulties) among 3 groups (normal, borderline, and clinical).

| Group (number) | BPA | MnBP | MiBP | MEHP | MECPP | ∑DBP_m_ | ∑DEHP_m_ |
| --- | --- | --- | --- | --- | --- | --- | --- |
|  | OR (95% CI) | | | | | | |
| Normal (245) | Ref. | Ref. | Ref. | Ref. | Ref. | Ref. | Ref. |
| Borderline (125) | 1.30 (0.93, 1.81) | 0.42 (0.17, 1.06) | 0.31 (0.11, 0.87)+ | 0.80 (0.52, 1.23) | 0.80 (0.37, 1.73) | 0.39 (0.15, 1.01) | 0.79 (0.49, 1.25) |
| Clinical (88) | 1.22 (0.79, 1.87) | 0.84 (0.27, 2.66) | 0.68 (0.19, 2.43) | 1.13 (0.70, 1.84) | 2.04 (0.78, 5.30) | 0.79 (0.24, 2.61) | 1.18 (0.70, 2.01) |

Adjusted for parental ages, maternal cotinine levels, family income during pregnancy, child sex, birth order (first child or not), and child age at SDQ complete.

+p < 0.05

Table S5 Adjusted odds ratios for ten folds increase of maternal BPA and phthalate metabolite levels on having behavioral problems using 80% cutoff scores.

|  | Number of children in borderline/clinical (%) | BPA | MnBP | MiBP | MEHP | MECPP | ∑DBP_m_ | ∑DEHP_m_ |
| --- | --- | --- | --- | --- | --- | --- | --- | --- |
|  |  | OR (95% CI) | | | | | | |
| **Total difficulties (≧13)** | 88 (19.2) | 1.06  (0.72, 1.57) | 1.15  (0.38, 3.47) | 1.06  (0.31, 3.62) | 1.20  (0.76, 1.88) | 2.02  (0.84, 4.87) | 1.11  (0.35, 3.53) | 1.26  (0.77, 2.05) |
| **Conduct problems (≧4)** | 71 (15.5) | 1.03  (0.68, 1.57) | 0.77  (0.24, 2.43) | 0.69  (0.19, 2.48) | 0.85  (0.53, 1.37) | 2.98  (1.17, 7.59)^+^ | 0.74  (0.22, 2.46) | 0.89  (0.53, 1.49) |
| **Hyperactivity/inattention (≧6)** | 81 (17.7) | 1.02  (0.68, 1.53) | 1.20  (0.39, 3.69) | 1.11  (0.32, 3.80) | 1.49  (0.94, 2.36) | 1.53  (0.63, 3.72) | 1.17  (0.36, 3.76) | 1.54  (0.94, 2.55) |
| **Emotional symptoms (≧4)** | 98 (21.4) | 0.75  (0.51, 1.12) | 0.65  (0.24, 1.74) | 0.56  (0.19, 1.66) | 0.77  (0.50, 1.21) | 0.53  (0.24, 1.20) | 0.62  (0.22, 1.73) | 0.76  (0.47, 1.23) |
| **Peer problems (≧4)** | 64 (14.0) | 0.99  (0.65, 1.52) | 0.92  (0.30, 2.87) | 0.45  (0.14, 1.49) | 0.78  (0.47, 1.29) | 0.90  (0.36, 2.25) | 0.79  (0.25, 2.54) | 0.76  (0.44, 1.44) |
| **Prosocial behavior (≦5)** | 112 (24.5) | 1.46  (1.04, 2.06)+ | 0.97  (0.39, 2.40) | 0.96  (0.36, 2.56) | 0.89  (0.59, 1.33) | 1.10  (0.53, 2.31) | 0.95  (0.37, 2.42) | 0.88  (0.56, 1.36) |

Adjusted for parental ages, maternal cotinine levels, family income during pregnancy, child sex, birth order (first child or not), and child age at SDQ complete.

+p < 0.05
